# Recurrent *FOSL1* rearrangements in desmoplastic fibroblastoma

**DOI:** 10.1002/path.6038

**Published:** 2023-01-03

**Authors:** Solange De Noon, Robert Piggott, Jamie Trotman, John A Tadross, Matthew Fittall, Debbie Hughes, Hongtao Ye, Emani Munasinghe, Matthew Murray, Roberto Tirabosco, Fernanda Amary, Nicholas Coleman, James Watkins, Michael Hubank, Patrick Tarpey, Sam Behjati, Adrienne M Flanagan

**Affiliations:** ^1^ Research Department of Pathology University College London Cancer Institute London UK; ^2^ Department of Histopathology Royal National Orthopaedic Hospital Stanmore UK; ^3^ Cambridge Genomics Laboratory Cambridge University Hospitals NHS Foundation Trust Cambridge UK; ^4^ Department of Histopathology Cambridge University Hospitals NHS Foundation Trust Cambridge UK; ^5^ MRC Metabolic Diseases Unit, Wellcome Trust‐Medical Research Council Institute of Metabolic Science University of Cambridge Cambridge UK; ^6^ Department of Oncology University College London Hospitals NHS Foundation Trust London UK; ^7^ Division of Oncology University College London Cancer Institute London UK; ^8^ Paediatric Tumour Biology, Division of Clinical Studies The Institute of Cancer Research London UK; ^9^ Department of Paediatric Haematology and Oncology Cambridge University Hospitals NHS Foundation Trust Cambridge UK; ^10^ Department of Pathology University of Cambridge Cambridge UK; ^11^ Clinical Genomics The Royal Marsden NHS Foundation Trust London UK; ^12^ Molecular Pathology The Institute of Cancer Research London UK; ^13^ Cellular Genetics Wellcome Sanger Institute Hinxton UK; ^14^ Department of Paediatrics University of Cambridge Cambridge UK

**Keywords:** *FOSL1*, *FOS*, desmoplastic fibroblastoma, targeted sequencing, gene rearrangement

## Abstract

The FOS gene family has been implicated in tumourigenesis across several tumour types, particularly mesenchymal tumours. The rare fibrous tumour desmoplastic fibroblastoma is characterised by overexpression of FOSL1. However, previous studies using cytogenetic and molecular techniques did not identify an underlying somatic change involving the *FOSL1* gene to explain this finding. Prompted by an unusual index case, we report the discovery of a novel *FOSL1* rearrangement in desmoplastic fibroblastoma using whole‐genome and targeted RNA sequencing. We investigated 15 desmoplastic fibroblastomas and 15 fibromas of tendon sheath using immunohistochemistry, *in situ* hybridisation and targeted RNA sequencing. Rearrangements in *FOSL1* and *FOS* were identified in 10/15 and 2/15 desmoplastic fibroblastomas respectively, which mirrors the pattern of *FOS* rearrangements observed in benign bone and vascular tumours. Fibroma of tendon sheath, which shares histological features with desmoplastic fibroblastoma, harboured *USP6* rearrangements in 9/15 cases and did not demonstrate rearrangements in any of the four *FOS* genes. The overall concordance between FOSL1 immunohistochemistry and RNA sequencing results was 90%. These findings illustrate that *FOSL1* and *FOS* rearrangements are a recurrent event in desmoplastic fibroblastoma, establishing this finding as a useful diagnostic adjunct and expanding the spectrum of tumours driven by *FOS* gene family alterations. © 2022 The Authors. *The Journal of Pathology* published by John Wiley & Sons Ltd on behalf of The Pathological Society of Great Britain and Ireland.

## Introduction

The discovery of the *FOS* retroviral homologue (v‐fos) as an initiator of osteosarcoma in mice [1] spurred significant interest in the role of *FOS* and its paralogues in the pathogenesis of bone and other tumours. The *FOS* gene family comprises four genes, *FOS*, *FOSB*, *FOSL1* and *FOSL2*, which encode subunits of the activator protein 1 (AP1) transcription factor, a master regulator of human development and cellular differentiation [2, 3]. Cancer genomics efforts over the past decade have revealed that somatic rearrangements in *FOS* and *FOSB* underpin osteoblastoma [4], osteoid osteoma, epithelioid haemangioma [5, 6] and pseudomyogenic haemangioendothelioma [7]. Mutations of the remaining two *FOS* genes, *FOS Like 1* (*FOSL1*) and *FOS Like 2* (*FOSL2*), have not been demonstrated in human tumours to date. In particular, although the benign fibrous tumour desmoplastic fibroblastoma (collagenous fibroma) is characterised by FOSL1 overexpression [8], cytogenetic studies have localised recurrent breakpoints to chromosome 11q12, adjacent to but not involving the *FOSL1* gene [8, 9, 10].

Here we outline the initial discovery by whole‐genome sequencing of a *FOSL1* rearrangement that recapitulates the pattern of reported *FOS* variants in an index case of desmoplastic fibroblastoma. We further demonstrate that rearrangements in *FOSL1* and, less commonly *FOS* represent a recurrent and specific feature in the majority of these tumours.

## Materials and methods

### Whole‐genome sequencing (index case)

Fresh frozen tumour and whole blood were submitted for whole‐genome sequencing through the National Health Service (NHS) Genomic Medicine Service via the East Genomics Laboratory Hub (GLH), Cambridge, UK, as previously described [11]. Data processing and analysis were performed using the established clinical pipeline at the East GLH. All data presented for the index case were generated as part of routine clinical care. The child's legal guardians provided informed consent for publication of their child's case. The validation cohort was obtained through the University College London/University College London Hospitals (UCL/UCLH) Biobank for Health and Disease (REC reference 20/YH/0088).

### Targeted RNA sequencing

Archival formalin‐fixed paraffin‐embedded (FFPE) material from 15 cases each of desmoplastic fibroblastoma and fibroma of tendon sheath were obtained from the UCL/UCLH tissue biobank at the Royal National Orthopaedic Hospital (RNOH). RNA was extracted from tumour FFPE material and analysed using the TruSight® RNA Pan‐Cancer Panel (Illumina, San Diego, CA, USA) according to the manufacturer's protocol. This panel allows for targeted enrichment of the exonic sequences of 1,385 cancer‐related genes, including *FOS*, *FOSB*, *FOSL1* and *USP6*. Bioinformatic analysis was performed using the RNA‐Seq Alignment App version 2.0.1 (BaseSpace Sequencing Hub, Illumina) with default parameters (Supplementary materials and methods). This was followed by analysis using a second, clinically validated in‐house pipeline based on Arriba [12] at the North Thames GLH. Sequencing data were manually inspected for reads supporting breakpoints across the four *FOS* genes using the Integrative Genomics Viewer [13].

### Immunohistochemistry and FISH analysis

All samples were subjected to FOSL1 immunohistochemistry using Anti‐Fra‐1 (C‐12, Santa Cruz Biotechnologies, Texas, USA, see Supplementary materials and methods). c‐FOS immunohistochemistry was performed using Anti‐c‐Fos (ABE457, 0.5 μg/ml, MilliporeSigma, Burlington, MA, USA). Fluorescence *in situ* hybridisation (FISH) analysis for *USP6* breakpoints was performed on all fibromas of tendon sheath samples with commercially available *USP6* dual colour probe (Zytovision, Bremerhaven, Germany).

## Results

**Figure 1:**
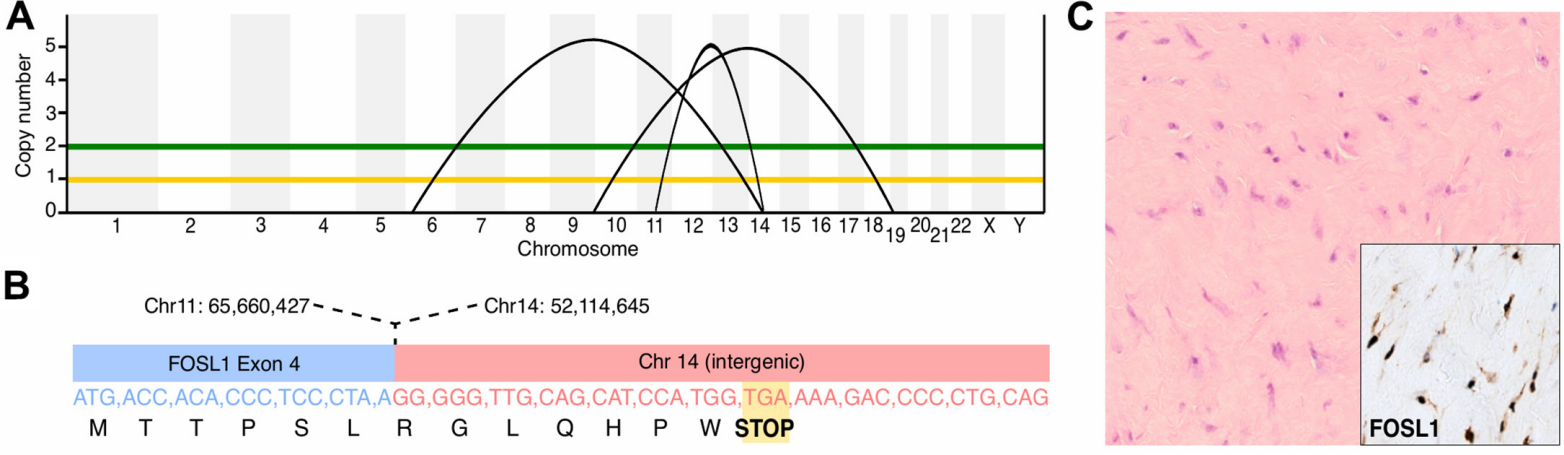
*FOSL1* rearrangement in index case. (A) Overview of somatic copy number and structural variant analysis of index tumour whole genome, including t(11;14)(q13.1;q22.1) rearrangement involving *FOSL1*. Green indicates absolute copy number, yellow minor allele copy number. (B) Schematic of *FOSL1* rearrangement. Transcript sequence shows introduction of premature stop codon. (C) Haematoxylin and eosin (H&E) showed a paucicellular fibrous lesion composed of bland spindle cells, which demonstrated strong nuclear immunoreactivity to FOSL1 (inset).

This study began with a case of an infant who had developed an infiltrative mass in the dorsal compartment of the distal forearm, near the wrist. This mass was initially noted during the first few weeks of life; progressive growth for several months prompted an open biopsy at a separate hospital. Histology demonstrated a bland, hypocellular spindle cell tumour with a myxocollagenous stroma, which lacked informative diagnostic features on immunohistochemistry. No convincing evidence of malignancy was observed, but a low‐grade sarcoma could not be excluded. Given surgical options for *en bloc* removal without functional impairment were limited, chemotherapy was proposed as is commonly employed in unresectable, low‐grade fibromatous tumours of childhood. The child then came under our care, at which time the growth of the lesion had stabilised. A decision was made to monitor the tumour closely before embarking on definitive treatment. Nine months later the mass increased in size, and the lesion was excised. At this time, the diagnosis of a benign fibrous tumour could be provided with greater confidence; the favoured differential diagnoses included a fibroma of tendon sheath and desmoplastic fibroblastoma (Figure 1C). No histological features of malignancy were identified.

Clinical whole‐genome sequencing, performed through the NHS Genomic Medicine Service, revealed a rearrangement involving the *FOSL1* gene on chromosome 11 (Figure 1A). The *FOSL1* breakpoint was located in the final exon of the gene, with the partner sequence belonging to an intergenic region on chromosome 14. The location of this breakpoint ostensibly disconnects the functional coding sequences from its terminal regulatory domain, mirroring the pattern of rearrangements previously described in *FOS* [4, 6]. The tumour genome was otherwise devoid of somatic copy number changes or point mutations that generated plausible driver events. Targeted RNA sequencing, performed on FFPE‐derived cDNA using TruSight® RNA Pan‐Cancer Panel, confirmed the *FOSL1* breakpoint at the transcript level (Figure 1B), which was further corroborated by strong nuclear immunoreactivity for FOSL1 (Figure 1C). Based on these findings, a final diagnosis of desmoplastic fibroblastoma was concluded, and we speculated that somatic *FOSL1* rearrangements may underpin desmoplastic fibroblastoma.

**Figure 2:**
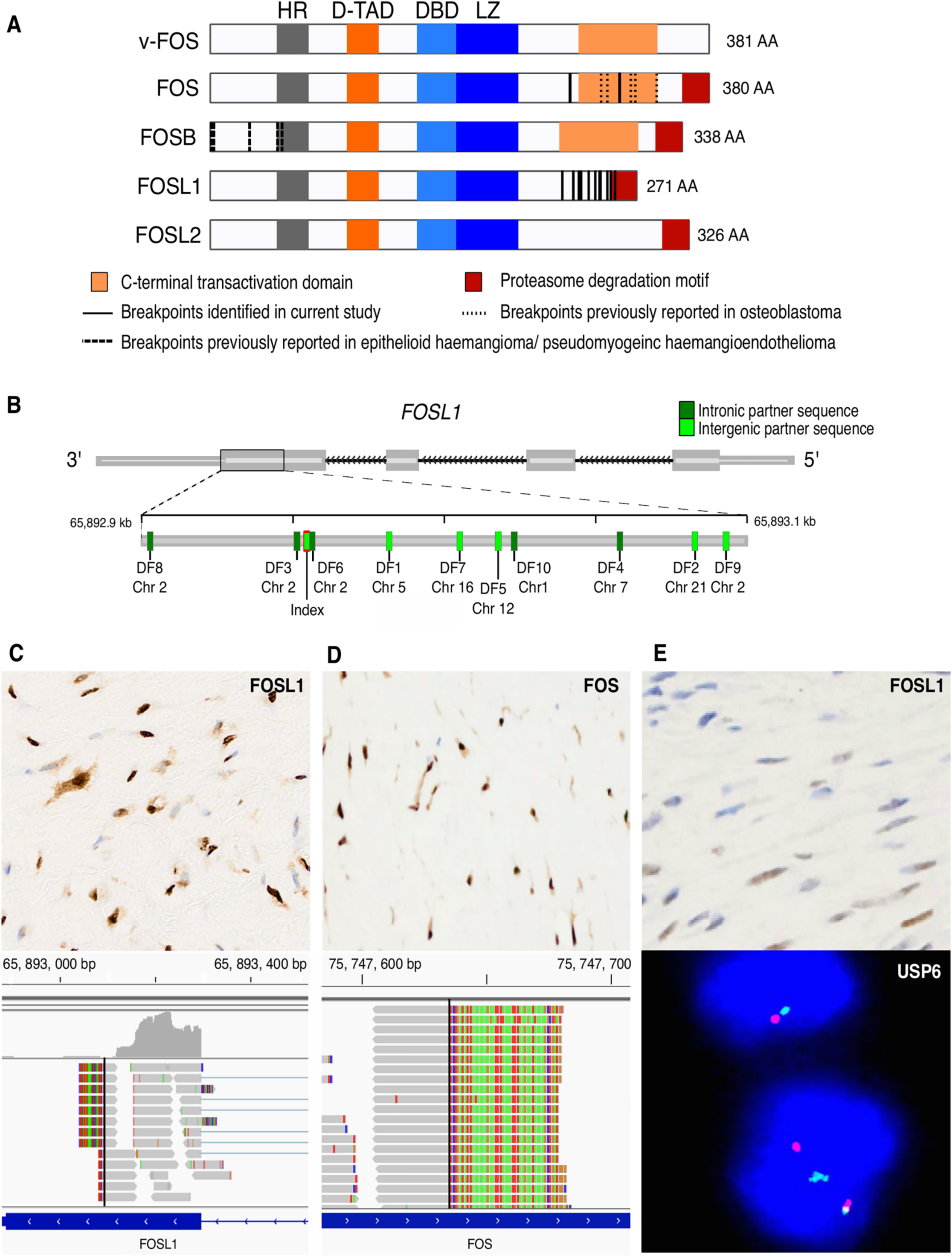
*FOSL1* and *FOS* rearrangements in desmoplastic fibroblastoma. (A) Schematic of *FOS* gene family transcripts showing shared pattern of breakpoints in *FOS* and *FOSL1*, leading to detachment of the proteasome degradation motif. HR, homologous region; D‐TAD, dominant transactivation domain; DBD, DNA binding domain; LZ, leucine zipper region. (B) Clustering of *FOSL1* breakpoints detected in desmoplastic fibroblastoma in exon 4 of gene. (C–E) Correlation of immunohistochemical and targeted RNA sequencing results. Strong diffuse immunoreactivity for (C) FOSL1 or (D) FOS were associated with the presence of rearrangements in the respective genes. (E) Weak and partial FOSL1 expression was seen in some fibromas of tendon sheaths, but typical *USP6* rearrangements and absence of *FOSL1* rearrangements distinguish these tumours.

We investigated 15 additional cases of desmoplastic fibroblastoma by targeted sequencing of FFPE‐derived cDNA and correlated the transcriptomic findings with immunohistochemistry for FOSL1 (supplementary material, Table S1). In a cohort of 15 desmoplastic fibroblastomas, we found strong FOSL1 immunopositivity in 12/15 cases (including one recurrent tumour DF10), 10 of which harboured *FOSL1* rearrangements at the transcript level (Figure 2, supplementary material, Table S2). The remaining 3/15 cases did not exhibit FOSL1 immunoreactivity or *FOSL1* rearrangements. Similar to the index case, all *FOSL1* breakpoints clustered around the regulatory domain of the final exon (Figure 2A,B). Rearrangement partners were scattered across multiple chromosomes, most commonly chromosome 2 (*n* = 4), and all comprised non‐coding regions, either intronic (out of reading frame, *n* = 5) or intergenic (*n* = 5). Despite strong FOSL1 expression in two cases, DF11 and DF12, these revealed no evidence of rearrangements in *FOSL1*.

We next delved into the three *FOSL1* wild‐type and immunonegative cases of desmoplastic fibroblastoma and found that two of these contained breakpoints in the final exon of *FOS*, identical to the recurrent alterations that typify osteoblastoma (Figure 2A). Consistent with this finding, we demonstrated c‐*FOS* immunoreactivity in both cases (Figure 2D). In the third *FOSL1* wild‐type case (DF15), an intrachromosomal *TFG–PIK3CA* translocation was identified.

To explore the specificity of these findings in *FOS* and *FOSL1* for desmoplastic fibroblastoma, we examined 15 fibroma of tendon sheath tumours, their principal histological mimic. As 90% of these contain USP6 rearrangements [14], we submitted all cases for FOSL1 immunohistochemistry and FISH for a *USP6* break‐apart signal. Nine fibromas of tendon sheath harboured *USP6* break‐apart signals (Figure 2E). FOSL1 immunostaining was negative in 12/15 cases, whereas 3/15 (FTS5, FTS11, FTS12) showed equivocal weak nuclear immunoreactivity (1+) with a minor population of tumour cells demonstrating stronger (2+/3+) positivity (Figure 2E). No fibromas showed strong uniform FOSL1 positivity, as observed in desmoplastic fibroblastoma. We interrogated the six fibromas of tendon sheath, which showed no *USP6* rearrangement by FISH, using targeted RNA sequencing, and did not find a breakpoint in *FOSL1* or any of the other three *FOS* genes. Across both tumour types, concordance between FOSL1 immunohistochemistry and targeted sequencing results was 90%. Together, these findings indicated that *FOSL1* rearrangements, detectable by immunohistochemistry or direct sequencing of the *FOSL1* transcript, are a common feature in desmoplastic fibroblastoma and absent from fibroma of tendon sheath, including *USP6* wild‐type cases.

## Discussion

Our investigation revealed that a majority of desmoplastic fibroblastomas harbour rearrangements in *FOSL1* that distinguish them from their main mimic, fibroma of tendon sheath. Although conventional morphology alone will suffice in most cases to reach a diagnosis of desmoplastic fibroblastoma, some rare cases will require more definitive evidence to aid clinical decision making, as illustrated by the index patient of this study. The finding of a molecular marker that confirmed the benign nature of this tumour‐directed clinical management to prioritise long‐term functional outcomes over tumour eradication. In addition, identification of *FOSL1* rearrangements has allowed confirmation of a case of locally recurrent desmoplastic fibroblastoma (DF10). Disease relapse has not been reported in the literature for this tumour type, so our finding expands the spectrum of clinical behaviour displayed by this entity.

Similar to the need for cautious interpretation of FOS and FOSB immunoreactivity [15, 16], careful optimisation of the dilution of antibodies against FOSL1 is required since wild‐type cells can demonstrate low levels of this protein [17]. Hence, detection of *FOSL1* rearrangements by targeted sequencing approaches may be the preferred adjunct for the diagnostic work‐up of fibrous tumours: this resolves the challenge of interpreting equivocal immunohistochemistry while simultaneously identifying genetic alterations that may indicate other fibrous tumours, including desmoid‐type fibromatosis, nodular fasciitis and low‐grade fibromyxoid sarcoma.

Earlier genetic studies of desmoplastic fibroblastoma identified somatic changes in the vicinity of the *FOSL1* locus, associated with FOSL1 immunoreactivity [9, 10]. Whole‐genome and RNA sequencing enabled us to unravel the underlying somatic genetic alteration that explained these findings. In a remarkable parallel to rearrangements observed in *FOS*, translocations remove the regulatory region of *FOSL1* to generate a mutant gene mimicking the potent oncogene v‐fos [4]. The regulatory region of *FOSL1* encodes motifs that are highly conserved across all *FOS* genes and promote protein degradation [18, 19, 20]. Disruption of these motifs is hypothesised to increase activity by increasing protein life span [6], which in the case of *FOS* has been corroborated experimentally *in vitro* [21]. It is thus highly plausible that *FOSL1* rearrangements operate through the same mechanism, i.e. reduced FOSL1 protein degradation, which is supported by our finding of intense FOSL1 protein immunoreactivity in mutant tumours.

Since systematic large‐scale efforts to investigate human neoplasms have concluded, the somatic genetic landscape of the majority of tumour types has been defined. Precision medicine programmes, such as whole‐genome sequencing offered to children with tumours and all patients with sarcoma, by the National Health Service in England [11] provide an opportunity to study genetically uncharted neoplasms in a real‐life clinical context. As three of four *FOS* genes have emerged as recurrently mutated in human tumours, and given the structural and functional similarities between members of this gene family, we suspect that there may well be neoplasms harbouring yet undiscovered alterations in *FOSL2*, which clinical sequencing programmes of rare tumours can help reveal.

## Author contributions statement

RP, JT, JAT, JW and PT performed sequencing and analysis of the index case. PT, DH, MH and SDN performed sequencing and analysis of the validation cohorts. SB and MF contributed to bioinformatic analyses. RT, FA, JW, MM and NC were involved in clinical and pathological interpretations. HY and EM performed FISH analysis. AMF and SDN identified the validation cohort and evaluated the immunohistochemical results. SDN wrote the manuscript, with contributions from DH, AMF and SB. AMF and SB directed this research.

## Supporting information


Supplementary materials and methods

**Table S1.** Summary of clinical demographics and immunohistochemical, FISH and targeted RNA sequencing results for validation cohort
**Table S2.** *FOSL1* and *FOS* breakpoints identified on targeted RNA sequencing

## Data Availability

The authors declare that all data supporting the findings of this study are available within the article and its supplementary information files or from the corresponding author on reasonable request.
