# Supplementary material for: Recurrent FOSL1 rearrangements in desmoplastic fibroblastoma
Source: J Pathol. 2023 Jan 3;259(2):119–24. doi: 10.1002/path.6038 (PMC10107450; doi:10.1002/path.6038)
Supplement: Supplementary file 1 — Supplementary materials and methods [file PATH-259-119-s001.docx]

**Recurrent *FOSL1* rearrangements in desmoplastic fibroblastoma**

S De Noon *et al. J Pathol* <https://doi.org/10.1002/path.6038>

**Supplementary materials and methods**

Reference numbers refer to the main text list.

Targeted RNA sequencing

RNA was extracted from tumour FFPE material, and samples meeting the minimum concentration and DV200 requirements were selected for targeted sequencing. RNA was analysed using the TruSight RNA Pan-Cancer Panel (Illumina, San Diego, CA, USA) targeting 1,385 genes, according to the manufacturer’s protocol. Paired end sequencing (2×75bp) generated a minimum of 3 million unique aligned reads, in line with supplier recommendations. Bioinformatic analysis was performed firstly using the RNA-Seq Alignment App version 2.0.1 (BaseSpace Sequencing Hub, Illumina) using STAR aligner and Manta for gene fusion calling with default parameters. A second analysis was performed using the Molecular Diagnostics Information Management System, an in-house pipeline using STAR aligner (to RefSeq Homo Sapiens GRCh38/Hg38) and fusion callers Arriba and StarFusion. Sequencing data were also manually inspected for reads supporting breakpoints across the four *FOS* genes using the Integrative Genomics Viewer (<https://software.broadinstitute.org/software/igv/>; Broad Institute).

FOSL1 and FOS immunohistochemistry

Immunohistochemistry was performed using a Leica BOND-III and BOND Polymer Refine Detection Kit (Leica Microsystems Ltd, Milton Keynes, UK) using antibodies to FOS (ABE457, 0.5 µg/ml, MilliporeSigma, Burlington, MA, USA, dilution 1:500) and FOSL1 (Anti-Fra-1, C-12, Santa Cruz Biotechnologies, Dallas, TX, USA, dilution 1:300, ER1–20 minutes before treatment), as previously described [16]. Immunostained slides were scored as follows: Positive = tumour cell nuclear staining 2+ to 3+ in at least 50% of the tumour, with background cell nuclei staining absent or focal 1+. Equivocal = tumour cell nuclear staining 1+ in >50% of tumour or focal 2+ nuclear staining (<50%). Negative = complete absence of tumour cell nuclear staining or focal 1+ staining in <50% of the tumour.

*USP6* FISH analysis

A commercially available *USP6* dual colour probe (Zytovision, Bremerhaven, Germany) was used this study. FISH was performed on paraffin sections, as described previously [22]. Signals in a minimum of 50 consecutive non-overlapping nuclei were counted. A result was considered positive when the ratio of target gene to centromeric/telomeric control region was >2 in at least 10% of nuclei.
